# Supplementary material for: Kv2.1 mediates spatial and functional coupling of L-type calcium channels and ryanodine receptors in mammalian neurons
Source: eLife. 2019 Oct 30;8:e49953. doi: 10.7554/eLife.49953 (PMC6839919; doi:10.7554/eLife.49953)
Supplement: Transparent reporting form [file elife-49953-transrepform.docx]

***eLife’s* transparent reporting form**

We encourage authors to provide detailed information *within their submission* to facilitate the interpretation and replication of experiments. Authors can upload supporting documentation to indicate the use of appropriate reporting guidelines for health-related research (see [EQUATOR Network](http://www.equator-network.org/%20)), life science research (see the [BioSharing Information Resource](https://biosharing.org/)), or the [ARRIVE guidelines](http://www.plosbiology.org/article/info:doi/10.1371/journal.pbio.1000412) for reporting work involving animal research. Where applicable, authors should refer to any relevant reporting standards documents in this form.

If you have any questions, please consult our Journal Policies and/or contact us: [editorial@elifesciences.org](mailto:editorial@elifesciences.org).

**Sample-size estimation**

- You should state whether an appropriate sample size was computed when the study was being designed
- You should state the statistical method of sample size computation and any required assumptions
- If no explicit power analysis was used, you should describe how you decided what sample (replicate) size (number) to use

Please outline where this information can be found within the submission (e.g., sections or figure legends), or explain why this information doesn’t apply to your submission:

We did not perform an explicit determination of appropriate sample size for most of the experiments presented within this study. However, for live cell experiments, based on our prior experimental experience and biostatistical analysis with *PS Power and Sample Size Calculation* software (Vanderbilt University, Nashville, TN) using the approach of Dupont and Plummer (1990) for paired samples, we had previously estimated that we would need to examine approximately 13 cells per condition to detect statistical differences between treatment conditions. This was assuming differences that were at least 50% of the control group mean, with a standard deviation approximately 25% of the mean, and assuming an α=0.05 and power=0.9. The n-values reported here are largely in-line with these predicted appropriate sample sizes. In some cases we observed very large differences between the control and treatment groups, so fewer cells were required to make a determination of statistical significance. Here, we presented the values from each individual cell as scatter plots, detailed the numbers of cells and mice used in each experiment contained in this study, and described the statistical test used to compare groups for each experiment in the figures and/or figure legends of each figure.

Dupont WD, Plummer WD: "Power and Sample Size Calculations: A Review and Computer Program", Controlled Clinical Trials 1990; 11:116-28.

**Replicates**

- You should report how often each experiment was performed
- You should include a definition of biological versus technical replication
- The data obtained should be provided and sufficient information should be provided to indicate the number of independent biological and/or technical replicates
- If you encountered any outliers, you should describe how these were handled
- Criteria for exclusion/inclusion of data should be clearly stated
- High-throughput sequence data should be uploaded before submission, with a private link for reviewers provided (these are available from both GEO and ArrayExpress)

Please outline where this information can be found within the submission (e.g., sections or figure legends), or explain why this information doesn’t apply to your submission:

This information is described in our materials and methods. Additionally, n numbers are reported in the figures and/or figure legends. For immunohistochemistry experiments involving mice, we define biological replicates as individual animals and have included biological replicates. For experiments involving cultured mammalian cells, we define technical replicates as experiments performed on the same day, and biological replicates as experiments performed on different days with different cultures. Our experiments with cultured mammalian cells contain biological and/or technical replicates. We did not exclude any data from this study. We did not utilize or generate high-throughput sequence data in this study.

**Statistical reporting**

- Statistical analysis methods should be described and justified
- Raw data should be presented in figures whenever informative to do so (typically when N per group is less than 10)
- For each experiment, you should identify the statistical tests used, exact values of N, definitions of center, methods of multiple test correction, and dispersion and precision measures (e.g., mean, median, SD, SEM, confidence intervals; and, for the major substantive results, a measure of effect size (e.g., Pearson's r, Cohen's d)
- Report exact p-values wherever possible alongside the summary statistics and 95% confidence intervals. These should be reported for all key questions and not only when the p-value is less than 0.05.

Please outline where this information can be found within the submission (e.g., sections or figure legends), or explain why this information doesn’t apply to your submission:

For all data presented in this manuscript, we have summarized n numbers, mean +/- standard error or standard deviation (stated in figure legend) and exact p-values reported in each figure or figure legend. Paired data sets were compared using a Student's t-test if the data passed a normality test; a non-parametric test was used otherwise. We used a one-way ANOVA followed by Dunnett’s post-hoc test to compare three or more groups, comparing the experimental group against the control data set in each experiment.

(For large datasets, or papers with a very large number of statistical tests, you may upload a single table file with tests, Ns, etc., with reference to sections in the manuscript.)

**Group allocation**

- Indicate how samples were allocated into experimental groups (in the case of clinical studies, please specify allocation to treatment method); if randomization was used, please also state if restricted randomization was applied
- Indicate if masking was used during group allocation, data collection and/or data analysis

Please outline where this information can be found within the submission (e.g., sections or figure legends), or explain why this information doesn’t apply to your submission:

For experiments involving animals, experimental groups were defined by genotype (i.e. wild-type vs. Kv2.1 knockout etc.). For experiments involving cell lines, experimental groups were defined by their transfected components (e.g., GFP-Cav1.2 alone vs. GFP-Cav1.2 + DsRed-Kv2.1). This information is apparent from the figures and/or figure legends.

**Additional data files (“source data”)**

- We encourage you to upload relevant additional data files, such as numerical data that are represented as a graph in a figure, or as a summary table
- Where provided, these should be in the most useful format, and they can be uploaded as “Source data” files linked to a main figure or table
- Include model definition files including the full list of parameters used
- Include code used for data analysis (e.g., R, MatLab)
- Avoid stating that data files are “available upon request”

Please indicate the figures or tables for which source data files have been provided:

We have elected to present our quantitative data in a manner such that individual data points can be examined, relative to the mean +/- standard error or deviation, and that representative images and movies exhibiting the reported phenotypes are shown.
